# GenomicDistributions: fast analysis of genomic intervals with Bioconductor

**DOI:** 10.1186/s12864-022-08467-y

**Published:** 2022-04-12

**Authors:** Kristyna Kupkova, Jose Verdezoto Mosquera, Jason P. Smith, Michał Stolarczyk, Tessa L. Danehy, John T. Lawson, Bingjie Xue, John T. Stubbs, Nathan LeRoy, Nathan C. Sheffield

**Affiliations:** 1 Center for Public Health Genomics, University of Virginia, Charlottesville, USA; 2 Department of Biochemistry and Molecular Genetics, University of Virginia, Charlottesville, USA; 3 Department of Biomedical Engineering, University of Virginia, Charlottesville, USA; 4 Department of Public Health Sciences, University of Virginia, Charlottesville, USA

**Keywords:** Genomic regions, Region set summary, Data visualization, R package, Bioconductor

## Abstract

**Background:**

Epigenome analysis relies on defined sets of genomic regions output by widely used assays such as ChIP-seq and ATAC-seq. Statistical analysis and visualization of genomic region sets is essential to answer biological questions in gene regulation. As the epigenomics community continues generating data, there will be an increasing need for software tools that can efficiently deal with more abundant and larger genomic region sets. Here, we introduce GenomicDistributions, an R package for fast and easy summarization and visualization of genomic region data.

**Results:**

GenomicDistributions offers a broad selection of functions to calculate properties of genomic region sets, such as feature distances, genomic partition overlaps, and more. GenomicDistributions functions are meticulously optimized for best-in-class speed and generally outperform comparable functions in existing R packages. GenomicDistributions also offers plotting functions that produce editable ggplot objects. All GenomicDistributions functions follow a uniform naming scheme and can handle either single or multiple region set inputs.

**Conclusions:**

GenomicDistributions offers a fast and scalable tool for exploratory genomic region set analysis and visualization. GenomicDistributions excels in user-friendliness, flexibility of outputs, breadth of functions, and computational performance. GenomicDistributions is available from Bioconductor (https://bioconductor.org/packages/release/bioc/html/GenomicDistributions.html).

**Supplementary Information:**

The online version contains supplementary material available at 10.1186/s12864-022-08467-y.

## Background

Sets of genomic regions are a fundamental data type for biological data analysis. They result from a variety of epigenome analysis experiments, such as ChIP-seq or ATAC-seq. Genomic region sets consist of genomic coordinates that specify regions with a shared property. Unlike genes, whose functions are better defined, the functional importance of non-coding genomic regions has been harder to interpret. To address this issue, multiple tools have been recently developed for a variety of analyses on genomic region sets, such as enrichment analysis (LOLA [1], LOLAweb [2], GIGGLE [3], IGD [4], GREAT [5], epiCOLOC [6]), visualization (chromPlot [7], karyoploteR [8]), region set comparison (BEDTools [9], Bedshift [10], AIList [11], regioneR [12]), or region annotation (Goldmine [13], annotatr [14], ChIPpeakAnno [15], ChIPseeker [16]). Other tools have been developed to classify and infer biological function of region sets (word2vec-based embedding [17], Avocado [18]), identify regulatory activity of regions (MIRA [19]), or to analyze heterogeneity across samples (COCOA [20]). Existing R packages provide some region-based analytical approaches, such as visualizing the distribution of genomic regions across chromosomes or annotations (chromPlot [7], karyoploteR [8], annotatr [14]), or for particular types of region sets, (e.g. ChIPpeakAnno [15], ChIPseeker [16] for ChIP-seq data), However, there is no general-purpose R package for extensive visualization and statistical analysis of genomic region sets from any source (Additional file 1: Table S1). Furthermore, many existing packages are not optimized to deal with the growing scale of region data, which now exceeds hundreds of thousands of publicly available region sets and hundreds of billions of individual regions [21–23].

**Fig. 1:**
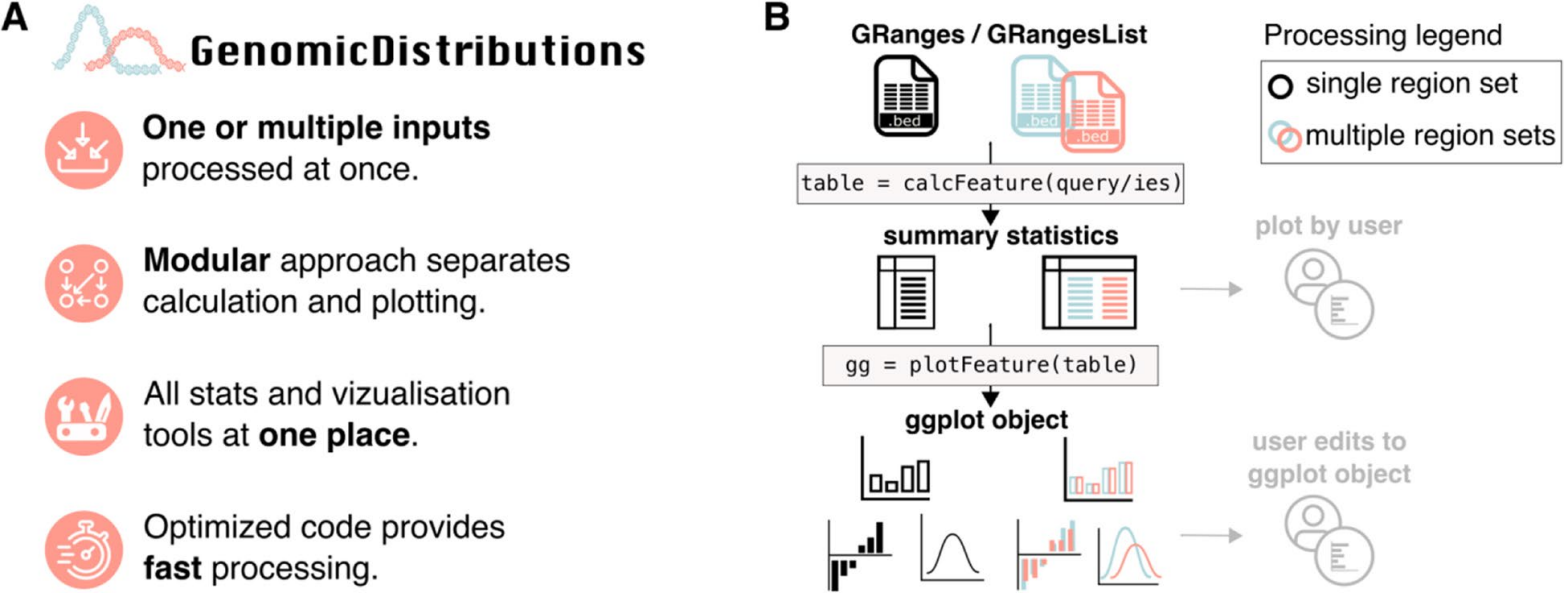
Overview and advantages of GenomicDistributions. **A** List of key design principles and advantages offered by GenomicDistributions. **B** GenomicDistributions functions are designed to process one or multiple genomic region sets at once. Plotting is separated from summary statistics calculation. Grey indicates that users may develop their own plots of summary statistics generated by *calc* functions, and edit ggplot objects from *plot* functions

To this end, we introduce the GenomicDistributions R package. GenomicDistributions specializes in computing basic statistics and visualizing distributions of genomic region sets from any experimental source. GenomicDistributions functions compute a variety of statistics and plot results to explore genomic region data. These include *chromosome distribution plots, feature distance plots, neighbor region distance plots, GC content plots, signal summary plots, genomic partition overlap plots*, *peak width quantile trimmed histogram plots,* and *dinucleotide frequency plots*. GenomicDistributions offers several key advantages over existing approaches (Fig. 1a): First, we carefully designed the package functions to have a simple, uniform, and flexible interface (Fig. 1b). GenomicDistributions functions all take the same input: a Bioconductor GenomicRanges or GenomicRangesList object, creating a unified interface for the user to summarize one or more region sets with the same line of code. We also separated calculation and plotting functions, enabling users to run calculations for reporting statistics without directly summarizing these only as plots. The outputs for every calculation function are the inputs for plotting functions. Furthermore, all plotting functions return ggplot2 objects, making it easier for users to adjust style of images. We also put considerable effort into optimizing performance, so GenomicDistributions scales better with large inputs than other R packages for related computations (Additional file 1: Fig. S1). Finally, GenomicDistributions provides a broad array of available analysis types. Its scope is more universal than many existing packages, targeting genomic interval data from any source (Additional file 1: Table S1).

## Results and discussion

GenomicDistributions functions calculate and plot summary statistics for single or multiple genomic region sets. These include functions for calculating distribution of regions across chromosomes, distances between neighboring regions, GC content, overlaps with genomic features, nearest distances from genomic features, widths of regions, and dinucleotide frequency, as well as functions for summary of user-provided signal values from different conditions.

Several functions require reference genome feature annotations. Users can either provide their own feature annotations or use wrapper functions (indicated with a “*Ref*” function name suffix), which require only a string specifying genome assembly as an input, and which automatically extract the feature annotations from the associated GenomicDistributionsData package. The wrapper functions are available for hg19, hg38, mm9, and mm10 reference genome assemblies; for other reference genomes, users can still use GenomicDistributions functions, but will need to supply the annotation data (Additional file 2: Table S2).

To demonstrate the use of GenomicDistributions, we put together a dataset of 5 genomic region sets of various types: EZH2 (Enhancer Of Zeste 2 Polycomb Repressive Complex 2 Subunit) regions in embryonic hepatocyte cells; FGF2 (Fibroblast Growth Factor 2) differentiation factor regions in iPSC (induced Pluripotent Stem Cells); and three sets of histone marks in B-cells, namely H3K27me3, which is associated with heterochromatin; H3K27ac, which is enriched in active enhancers; and H3K4me3, which is associated with active promoters. For this test set, we applied each GenomicDistributions calculation and plotting function. Here, we show examples of the resulting plots.

**Fig. 2:**
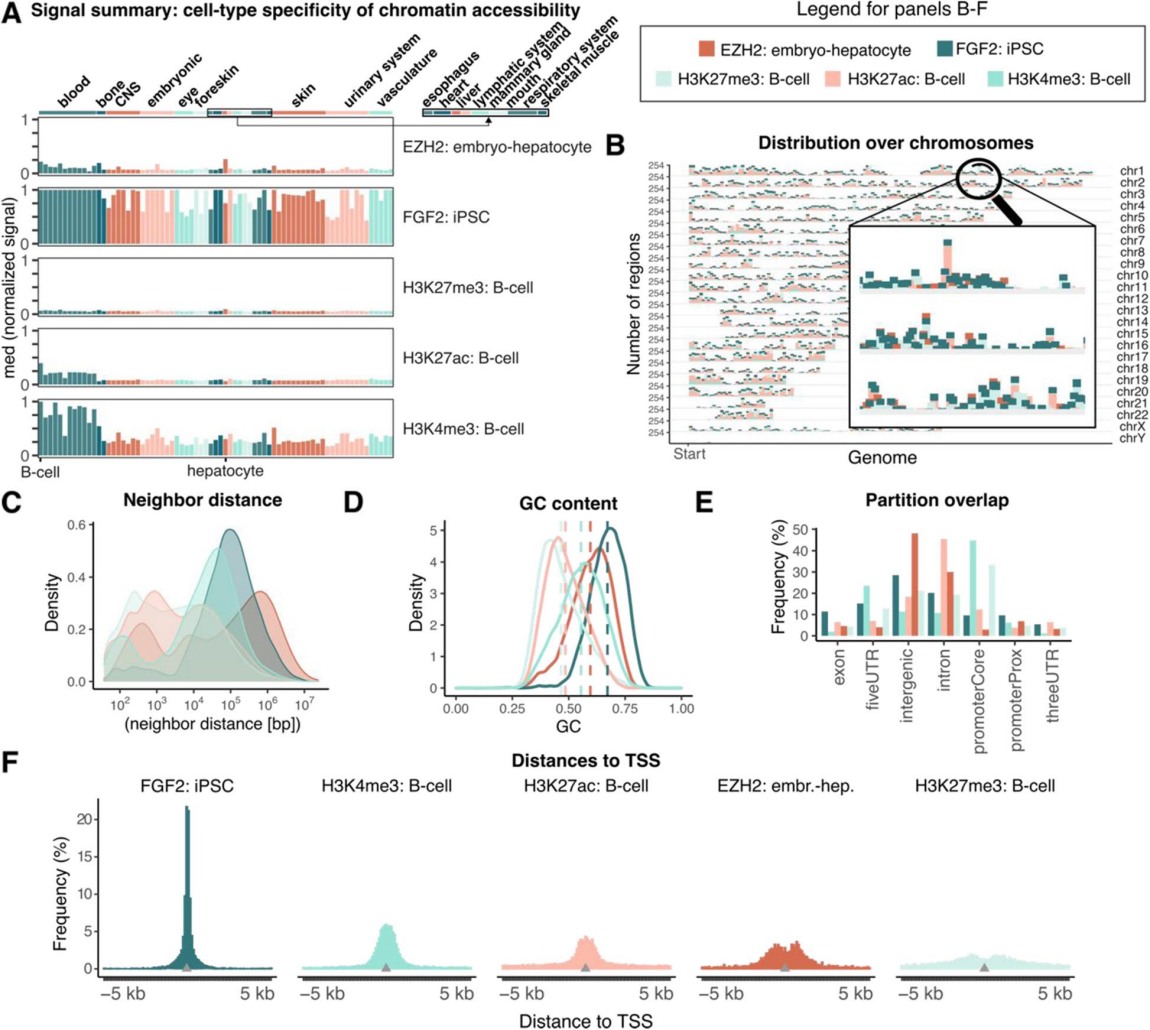
Example plots produced by GenomicDistributions. **A** Signal summary used for cell-type specificity of chromatin accessibility plot. Bars represent median values across regions. **B** Distribution of regions over chromosomes. **C** Distances between neighbor regions. **D** GC content in query regions. The dashed line indicates medians. **E** Partition distribution plot. **F** Distances to TSS

First, the signal summary function summarizes external signal data across query regions. This function requires a user-provided matrix with normalized signal values across a genome. GenomicDistributionsData provides a pre-constructed matrix of normalized chromatin accessibility signal values across genomes (hg19, hg38, mm10) of different cell types, which can be used to infer cell-type specificity of chromatin accessibility in the query regions (Fig. 2a). The output represents a summary of chromatin accessibility signal within test regions across different cell types. Next, the chromosome distribution plot (Fig. 2b) helps to visualize how the regions are distributed across chromosomes. The neighbor region distance plot (Fig. 2c) shows the distribution of distances between two consecutive regions in a sorted region set. The GC content plot (Fig. 2d) displays the distribution of GC content percentage within genomic regions of interest. The genomic partition distribution plot (Fig. 2e) shows how regions are distributed across genome annotation classes. Users can either provide features of interest or use the *calcPartitionsRef* function with pre-defined elements including core promoters, proximal promoters, exons, introns, 5′ UTRs (untranslated regions), 3′ UTRs, and intergenic regions (Additional file 2: Table S2). In addition to raw partition distributions plots (Fig. 2e), GenomicDistributions also offers expected partition distribution plots (Additional file 1: Fig. S2). Expected partition distribution plots correct the raw overlap counts (observed) by dividing those by expected overlaps, which depend on the size of the partition. This correction is particularly important, since the sizes of individual partitions such as exons vs. introns are considerably different, and therefore we expect more regions to overlap introns than exons by chance. The corrected overlap values are presented as the log_10_(observed/expected) overlap count for each partition. We then use the Chi-square independence test to calculate the *p*-values inferring the significance of the observed overlaps compared to expected (Additional file 1: Table S3). GenomicDistributions also provides a novel plot type that further extends this concept, called cumulative partition distribution plots (Additional file 1: Fig. S3, Supplementary methods). The cumulative partition distribution plot extends this concept in two ways: 1) it shows not only the total counts, but how they accumulate in total genome coverage when regions are ordered by size; and 2) instead of the fraction of regions in each feature, it shows a combined enrichment score, which is the average of the fraction of regions in each feature and the fraction of the features covered by regions. These two changes make the plots more informative, as they include the total size of bases covered in each fraction, and a more balanced enrichment score that naturally accounts for the different total coverage of each partition (Additional file 1: Fig. S3, Supplementary methods).

Another plot produced by GenomicDistributions is the feature distance plot, which shows how the regions are distributed with respect to the nearest feature of interest. Due to the common use of distances to nearest transcription start site (TSS), we provide the *calcTSSDistanceRef* function for convenience (Fig. 2f). The width distribution plot (Additional file 1: Fig. S4) shows widths of genomic regions as a histogram with clipped top percentiles, a feature that allows enhanced visual comparison by eliminating long tails. Finally, dinucleotide frequency plots (Additional file 1: Fig. S5) shows the distribution of dinucleotide content within genomic regions of interest.

Our design philosophy for GenomicDistributions included several key concepts that provide advantages over other tools with similar purpose: First, we separated calculation functions from plotting functions (Fig. 1b). While many visualization-based analysis functions run calculations and plotting at the same time (Additional file 1: Table S1), by decoupling them, GenomicDistributions provides the flexibility to use the two independently so the intermediate results can be used for other purposes in downstream tools. Each analysis is thus done in two steps: first, by calling a *calc* function, which returns a summarized set of computed statistics; and second, by passing this result to a *plot* function.

We also designed a consistent user interface for GenomicDistributions functions. Each *calc* function can accept either a GenomicRanges object representing a single set of intervals, or a GenomicRangesList object with multiple sets of intervals (Fig. 1a, b). This provides a single interface for either individual region set exploration or to compare among region sets. The result of all *calc* functions can then be used as direct input into a corresponding *plot* function. In turn, each *plot* function returns a ggplot object which can then be further styled by the user. This consistency across functions makes it simple to learn how to use the package and to run multiple analyses on a single input.

Most of the *plot* functions offer multiple plotting options. For example, the feature distance distribution plot offers: 1) the default histogram option (Fig. 2f); and 2) a heatmap option (Additional file 1: Fig. S6a). Similarly, the signal summary plot can be visualized as a bar plot with any defined groups included (Fig. 1a), or, for example, as a violin plot showing a subset of predefined groups, such as tissues or cell types (Additional file 1: Fig. S6b). In addition to the carefully designed user interfaces, we also took considerable effort to optimize GenomicDistributions for speed. By leveraging the highly optimized code in the R data.table package [24] and using fast rolling joins, GenomicDistributions calculation functions are much faster than alternatives in other R packages.

To showcase the speed efficiency and scalability of GenomicDistributions, we carried out a running time benchmark against relevant R packages with related functions (Additional file 1: Table S1)**.** Specifically, we tested the speed of four analyses: 1) distribution of regions across genomic partitions, 2) distance of regions to TSSs 3) distribution of regions across chromosomes and 4) distance of regions to user-defined features. To perform this benchmark, we assembled a collection of six ChIP-seq region sets displaying variability in terms of region number (less than 10,000 to more than 300,000 regions) and region widths. Narrow regions are represented by transcription factor region sets (TCF12: Transcription Factor 12, ATF3: Activating Transcription Factor 3; MEF2C: Myocyte Enhancer Factor 2C), while broader regions originate from histone mark region sets (for region set details see Additional file 1: Table S4, Supplementary methods). The running time of GenomicDistributions *calc/plot* functions is generally lower, and often much lower, than competing packages (Additional file 1: Fig. S1)**.** Furthermore, GenomicDistributions tends to scale much better with increasing number of regions.

## Conclusions

GenomicDistributions is an R package with a broad set of functions available in one place with the purpose to explore genomic regions. While other currently available tools provide some of the summary functions, GenomicDistributions is the most feature-rich, in terms of the number and type of statistics/plots that are produced. GenomicDistributions also takes a step forward on ease-of-use through our modular, consistent programming design. Multiple region sets can be analyzed as easily as a single one, and for common reference genomes, GenomicDistributions can make use of our pre-compiled genome annotations. When more flexibility is needed, GenomicDistributions is also adaptable enough to be used with any reference assembly. Not only can users provide their own annotations, or features of interest, we also give them the freedom to visualize the results in their own way either by using the output of *calc* functions, or by editing ggplot objects returned by *plot* functions. Lastly, GenomicDistributions is substantially faster and more scalable than other publicly available R packages. Together, these strengths make GenomicDistributions a fast, flexible, powerful, and easy-to-use package for analysis of genomic region set data.

## Methods

The package is available from https://bioconductor.org/packages/release/bioc/html/GenomicDistributions.html, or https://github.com/databio/GenomicDistributions. Detailed methods description is available in Additional file 1: Supplementary methods.

## Supplementary Information

**Additional file 1.** Contains supplementary methods, supplementary Figs. (S1-S6), supplementary Tables (S1, S3 and S4) and supplementary references.

**Additional file 2: Supplementary Table S2.**

## Data Availability

The test dataset was obtained from ENCODE [23] (accession numbers: ENCFF869UBZ, ENCFF539PUL, ENCFF969HEX), and CISTROME Data Browser [21, 22] (GEO accession numbers: GSM2698625, GSM2439179). The benchmark region sets were obtained from ENCODE (accession numbers: ENCFF264OCQ, ENCFF543TAQ, ENCFF145FYQ, ENCFF647QUI, ENCFF720VWD, ENCFF130BPH).

## Abbreviations

EZH2: Enhancer of Zeste 2 Polycomb Repressive Complex 2 Subunit
FGF2: Fibroblast Growth Factor 2
H3K27me3: Histone H3 lysine 27 trimethylation
H3K27ac: Histone H3 lysine 27 acetylation
H3K4me3: Histone H3 lysine 4 trimethylation
H3K4me1: Histone H3 lysine 4 monomethylation
iPSC: Induced Pluripotent Stem Cells
TSS: Transcription start site
UTR: Untranslated regions
TCF12: Transcription Factor 12
ATF3: Activating Transcription Factor 3
MEF2C: Myocyte Enhancer Factor 2C
hESC: Human Embryonic Stem Cell
