# Supplementary material for: GenomicDistributions: fast analysis of genomic intervals with Bioconductor
Source: BMC Genomics. 2022 Apr 12;23:299. doi: 10.1186/s12864-022-08467-y (PMC9003978; doi:10.1186/s12864-022-08467-y)
Supplement: Supplementary file 1 — Additional file 1. Contains supplementary methods, supplementary Figs. (S1-S6), supplementary Tables (S1, S3 and S4) and supplementary references. [file 12864_2022_8467_MOESM1_ESM.docx]

Supplementary material for “GenomicDistributions: fast analysis of genomic intervals with Bioconductor”

Table of Contents

[Supplementary methods 1](#_Toc97638469)

[Signal summary function (with cell-type specificity of chromatin accessibility) 1](#_Toc97638470)

[Cell specific chromatin accessibility matrix 2](#_Toc97638471)

[Chromosome distribution plot 2](#_Toc97638472)

[Neighbor distances 3](#_Toc97638473)

[GC content 3](#_Toc97638474)

[Partition plots 3](#_Toc97638475)

[Cumulative partition plots 4](#_Toc97638476)

[Distance to TSS or to user defined features 5](#_Toc97638477)

[Quantile trimmed width distribution plots 5](#_Toc97638478)

[Dinucleotide frequency plot 5](#_Toc97638479)

[GenomicDistributions running time benchmark 5](#_Toc97638480)

[Test datasets 6](#_Toc97638481)

[Supplementary figures 7](#_Toc97638482)

[Supplementary tables 13](#_Toc97638483)

[Supplementary references 15](#_Toc97638484)

# Supplementary methods

## Signal summary function (with cell-type specificity of chromatin accessibility)

We created *calcSummarySignal* and *plotSummarySignal* functions to calculate and plot summary of signal values under regions of interest. The *calcSummarySignal* function requires an input matrix, with rows corresponding to pre-defined regions (spanning whole genome), columns corresponding to signals data sets, such as cell types or conditions, and values representing normalized signal of interest, such as chromatin accessibility signal from ATAC-seq experiments. As a practical example, we provide a matrix of normalized chromatin accessibility across different cell types in the GenomicDistributionsData package. We describe the matrix construction in the next section. The function *calcSummarySignal* finds overlaps between query regions and regions defined in the matrix and returns a list containing two data.table objects: *signalMatrix*, and *matrixStats*. *SignalMatrix* contains normalized signal values from the input matrix overlapping with query regions. If a query region overlaps with more than one region in the input matrix, a mean of the values is returned. *MatrixStats* contains a *boxplot.stats* summary for each combination of query region set and signal column. The box plot summary consists of 5 values: the lower whisker, the lower ‘hinge’, the median, the upper ‘hinge’ and the extreme of the upper whisker.

## Cell specific chromatin accessibility matrix

For use with the *calcSummarySignal* function, to allow users to summarize chromatin accessibility across cell types for a query region set, we created matrices with normalized chromatin accessibility values across different cell types for hg19, hg38, and mm10 reference genome annotations. For each genome annotation we obtained bigWig files from The Encyclopedia of DNA elements (ENCODE) (1) by applying the following filters: organism: hg19, hg38, mm10 separately / assay type: chromatin accessibility / biosample classification: primary cell / available file types: bigWig. If a given cell type had more than one representative bigWig file, we merged those with UCSC *bigWigMerge* tool (2,3) with default parameters, followed by conversion of the bedGraph output back to bigWig with UCSC *bigWigToBedGraph* (2,3), also with default parameters.

To create a reference set of regions we downloaded chromatin accessibility regions across different cell types from ENCODE by applying following filters: organism: hg19, hg38, mm10 separately / assay type: chromatin accessibility / biosample classification: primary cell / available file types: bed narrowPeak. We then merged downloaded BED files into a single reference BED file with bedtools (4) *merge* function with default parameters.

We mapped the bigWig files onto the reference set of regions with UCSC *bigWigAverageOverBed* (2,3) function with -minMax option. We then used the mean values to create a matrix, where each row is a region from the reference region set, each column is a cell type, and each value is a mean coverage over the region in each cell type. We normalized the matrix by setting all signal values above 99^th^ percentile for each cell type to 1 and normalizing the values below 99^th^ percentile to fall into 0-1 range. After that we quantile-normalized the resulting table to make it more suitable for comparison among different cell types and rounded the values to 4 decimal places to reduce the size of the matrix.

## Chromosome distribution plot

To calculate and plot distribution of regions across chromosomes, we created *calcChromBins, calcChromBinsRef*, and *plotChromBins* functions. The *calcChromBins* function calculates the number of regions that fall into user-defined bins, which are provided to the function as a GRangesList. This function provides a user more flexibility to specify regions to calculate distribution over. The *calcChromBinsRef* wrapper function requires only a string specifying reference assembly and optional number of bins to divide chromosomes into. The bins are created automatically by extracting chromosome sizes from the GenomicDistributionsData package and dividing them into desired number of bins (default, 3000 bins). We obtained the chromosome sizes in GenomicDistributionsData package by using *seqlengths* function from *the* GenomeInfoDb package on the following BSgenome objects: *BSgenome.Hsapiens.UCSC.hg19, BSgenome.Hsapiens.UCSC.hg38, BSgenome.Mmusculus.UCSC.mm9, BSgenome.Mmusculus.UCSC.mm10*, which make the *calcChromBinsRef* function currently available for hg19, hg38, mm9, and mm10 reference assemblies.

## Neighbor distances

To calculate and plot distances between consecutive regions in a query region set, we created *calcNeighborDist* and *plotNeighborDist* functions. The *calcNeighborDist* calculates the distance from the end of one region to the start of the consecutive region. Distances of a region to upstream/downstream neighbors are calculated by chromosome after sorting the region sets. Neighboring distances are then grouped into a single numeric vector for visualization of their distribution. If two consecutive regions overlap, their distance is set to 0.

## GC content

The functions that calculate and plot GC content in query/queries are *calcGCContent*, *calcGCContentRef*, and *plotGCContent*. The *calcGCContent* function requires a BSgenome object provided by user, which can be automatically retrieved with *calcGCContentRef*, where the user provides a string input specifying genome assembly (built-in available for hg19, hg38, mm9, and mm10). GC content within query regions is then calculated with use of *alphabetFrequency* function from *Biostrings* package.

## Partition plots

A common region set analysis is to classify each region by genomic feature, such as promoters/exons/introns/etc, and then tabulate the frequency in a region set. To do this, we created *calcPartitions*, *calcPartitionsRef*, and *plotPartitions* functions. The *calcPartitions* function offers a flexible option for the user to define features as a list containing a GRanges object for each feature. These must be sorted from highest to lowest priority order. The partition distribution plots use mutually exclusive feature annotations using a priority list of partition annotations. We use the term partition to indicate that the query regions are annotated mutually exclusively, by priority. Therefore, once a region is classified as a feature with higher priority, it will not be tested for overlaps with features with lower priorities. *calcPartitionsRef* uses pre-computed partition annotations provided by GenomicDistributionsData, which are derived from public sources for common genomes in the order of core promoter, proximal promoter, 5’ untranslated region (5’UTR), 3’ untranslated region (3’UTR), exon, and intron. Annotations were obtained from EnsDb.Hsapiens.v86, EnsDb.Hsapiens.v75, EnsDb.Mmusculus.v79 packages for hg38, hg19, and mm10 reference assembly features. Any query regions that are not covered by a known partition are classified as intergenic by default. These functions also allow a user to provide a custom partition list and change the classification of the remaining regions.

We calculate expected feature overlap for *calcExpectedPartitions,* *calcExpectedPartitionsRef* based on proportional number of base pairs (bp) by which each feature contributes to genome (total number of bp on each feature divided by genome size). We then multiply the proportion of each feature by the total number of regions in each query region set, which then gives us an expected number of overlaps for each feature if query regions were uniformly distributed across the genome. Plotting is then done with *plotExpectedPartitions*, which plots log_10_(observed/expected) values, therefore positive values are higher than expected and negative lower than expected. In addition, the significance of the enrichment across each partition is calculated through a Chi-square test of independence by creating contingency tables for each partition (regions are categorized as observed/expected and overlapping/non-overlapping). Chi-square *p*-values denote whether the observed overlap frequencies were significantly different from the expected (defined based on how much each individual partition contributes to overall genome size). Chi-square *p*-values are returned along the number of observed and expected overlaps for each partition when calling the *calcExpectedPartitionsRef* function*.* Additionally, they can be optionally displayed on top of each partition by the *plotExpectedPartitions* function in the form of asterisks to avoid crowding when analyzing multiple region sets.

## Cumulative partition plots

We extended the above analysis of genomic partitions with novel functions for calculating and plotting what we call the “cumulative partition distribution.” These functions are *calcCumulativePartitions* (for user provided features), *calcCumulativePartitionsRef* (pre-compiled features), and *plotCumulativePartitions*. The cumulative partition distribution plot extends the above concepts in two ways: 1) instead of total raw or expected counts, it considers how they accumulate in total genome coverage when regions are ordered by size; and 2) instead of the fraction of regions in each feature, it shows a combined enrichment score, which is the average of the fraction of regions in each feature and the fraction of the features covered by regions. These two changes make the plots more informative, as they include the total size of bases covered in each fraction, and a more balanced enrichment score that naturally accounts for the different total coverage of each partition.

To calculate these statistics, we first sort the query regions by size. We then assign each region to the highest priority partition by computing overlap. For each partition, we compute the cumulative sum of regions in a partition divided by the number of query regions. We weight this calculation by the fraction of the partition covered by the region, so that regions with less overlap contribute proportionally less. We also calculate a complementary cumulative score: the proportion of the partition that is covered by query regions. This gives us two complementary scores: the first measures how enriched the user regions are across partitions, and the second measures how much of the partition is covered by the user regions. The final "enrichment score" is the geometric mean of these two ratios. This enrichment score provides a balanced, interpretable score for how a user set is distributed across genomic partitions. It naturally controls for the imbalance in sizes of partitions. To also show how query regions relate to total genome space covered, we plot the above cumulative enrichment score against the cumulative sum of bases in each feature. Thus, each partition also shows the absolute number of bases covered, which provides a further perspective on the plot.

*Interpreting cumulative partition plots:* In the cumulative partition plots, each partition is represented by a different curve. The height of the curve indicates the enrichment score, described above; briefly, it is a balanced score that averages how much of the query covers a genomic partition, and how much of the genomic partition is covered by the query. The final y-axis value of each curve can be interpreted as how much that partition is enriched in the query regions. The position of the curve on the x-axis indicates the overall bases covered by the partition; therefore, partitions that cover more of genome will arise further to the right. This allows the user to simultaneously visualize the enrichment (in the y-axis) as well as the total overall coverage (in the x-axis). Finally, the slope of the curve reflects the size of the regions. If coverage for a particular partition is driven by many small regions, then the slope will be steep; if coverage is driven by few large regions, then slope will be less steep. This is because the enrichment score (y-axis) accumulates with the count of regions, while the bases covered (x-axis) accumulates with the size of regions. Larger regions accumulate size more quickly than count, while smaller regions accumulate count more quickly than size. Therefore, partitions covered by fewer larger overlaps will have less slope than partitions covered by many smaller overlaps.

##

## Distance to TSS or to user defined features

The set of functions calculating and plotting the distance between query regions to the nearest TSS, or nearest feature set defined by user are following: *calcFeatureDistRefTSS*, *calcFeatureDist*, and *plotFeatureDist*. In *calcFeatureDistRefTSS* we built TSS indexes for hg19, hg38, mm9, and mm10 reference genomes from EnsDb.Hsapiens.v75, EnsDb.Hsapiens.v86, TxDb.Mmusculus.UCSC.mm9.knownGene , and EnsDb.Mmusculus.v79 packages respectively, These are part of GenomicDistributionsData package. The *calcFeatureDistRefTSS* function then calculates the distance from the mid-points of the query regions to the nearest TSSs. The function returns the downstream distances as negative values. The *calcFeatureDist* function works on the same principle, it only calculates the closest distances between query mid-point and mid-points of user-provided features.

## Quantile trimmed width distribution plots

To calculate and plot widths of query regions, we created *calcWidth*, and *plotQTHist* functions. The *calcWidth* function simply calculates widths of query regions in bp. Plotting these in form of regular histogram or density function introduces long tails. Therefore, we created *plotQTHist* function, which groups bottom and top quantiles (2% default) into a single bin, which creates a more easily interpretable visual representation.

## Dinucleotide frequency plot

The functions that calculate and plot dinucleotide frequency in queryregions are *calcDinucl*, *calcDinuclFreqRef,* and *plotDinuclFreq*. The *calcDinucl* function requires a BSgenome object provided by user, which can be automatically retrieved within *calcDinuclFreqRef* function, where user provides a string input specifying genome assembly (currently available for hg19, hg38, mm9, and mm10). Dinucleotide frequency content within query regions is then calculated with use of *dinucleotideFrequency* function from the Biostrings package.

## GenomicDistributions running time benchmark

To compare GenomicDistributions performance with comparable functionality from other packages, we assembled a collection of six ChIP-seq region sets that vary in both region number and width. We denoted region sets as “Small” if they contained fewer than 10,000 regions. Region sets were denoted as “Medium” if they contained 10,000-50,000 regions. Region sets were denoted as “Large” if they had more than 100,000 regions. To vary region width, half of the region sets are transcription factor (TF) ChIP-seq data and represent narrow regions, while the other half are histone ChIP-seq data that represent broader regions (see supplemental Additional file1: Table S3). Our comparison focused on 4 analyses: 1) distribution of regions over genomic partitions, comparing GenomicDistributions, ChIPseeker, ChIPpeakAnno, annotatr, and Goldmine; 2) distance to TSS, comparing GenomicDistributions, ChIPseeker, and ChIPpeakAnno; 3) distribution over chromosomes, comparing GenomicDistributions, karyoploteR, chromPlot, and ChIPseeker; and 4) distance to user-defined features, comparing GenomicDistributions and ChIPpeakAnno. The running time for each package was measured using the R package microbenchmark v1.4.9 (5). GenomicDistributions uses data.table for fast analysis. To control for the implicit multi-threading feature of data.table during the running time benchmark, we restricted data.table to using a single thread (Fig. S1). Number of threads were set using the data.table function *setDTthreads*. The system in which the benchmark was performed had an *omp_get_num_procs* parameter of 16 (number of threads used cannot exceed this value).

## Test datasets

We obtained our representative datasets from ENCODE (1) and CISTROME (6,7) databases. The histone mark datasets originate from ENCODE database, and are available under following accession numbers: H3K4me3:B-cell - ENCFF869UBZ / H3K27me3:B-cell - ENCFF539PUL / H3K27ac:B-cell - ENCFF969HEX. The transcription factor BED files originate from CISTROME database and can be found under following GEO accession numbers: EZH2:embryo-hepatocyte - GSM2698625 / FGF2:iPSC - GSM2439179.

The region sets used for the running time benchmark were obtained from the ENCODE (1) database and are available under the following accession numbers: TCF12 ChIP-seq: human H1-hESC cell line - ENCFF264OCQ / H3K27me3: H9-hepatocyte - ENCFF543TAQ / ATF3 ChIP-seq: K562 cell line - ENCFF145FYQ / H3K4me3 ChIP-seq: tibial artery tissue (male adult; 37 years) - ENCFF647QUI / MEF2C ChIP-seq: GM12878 cell line – ENCFF720VWD / H3K4me1 ChIP-seq: GM23338 bipolar neuron – ENCFF130BPH.

#

# Supplementary figures


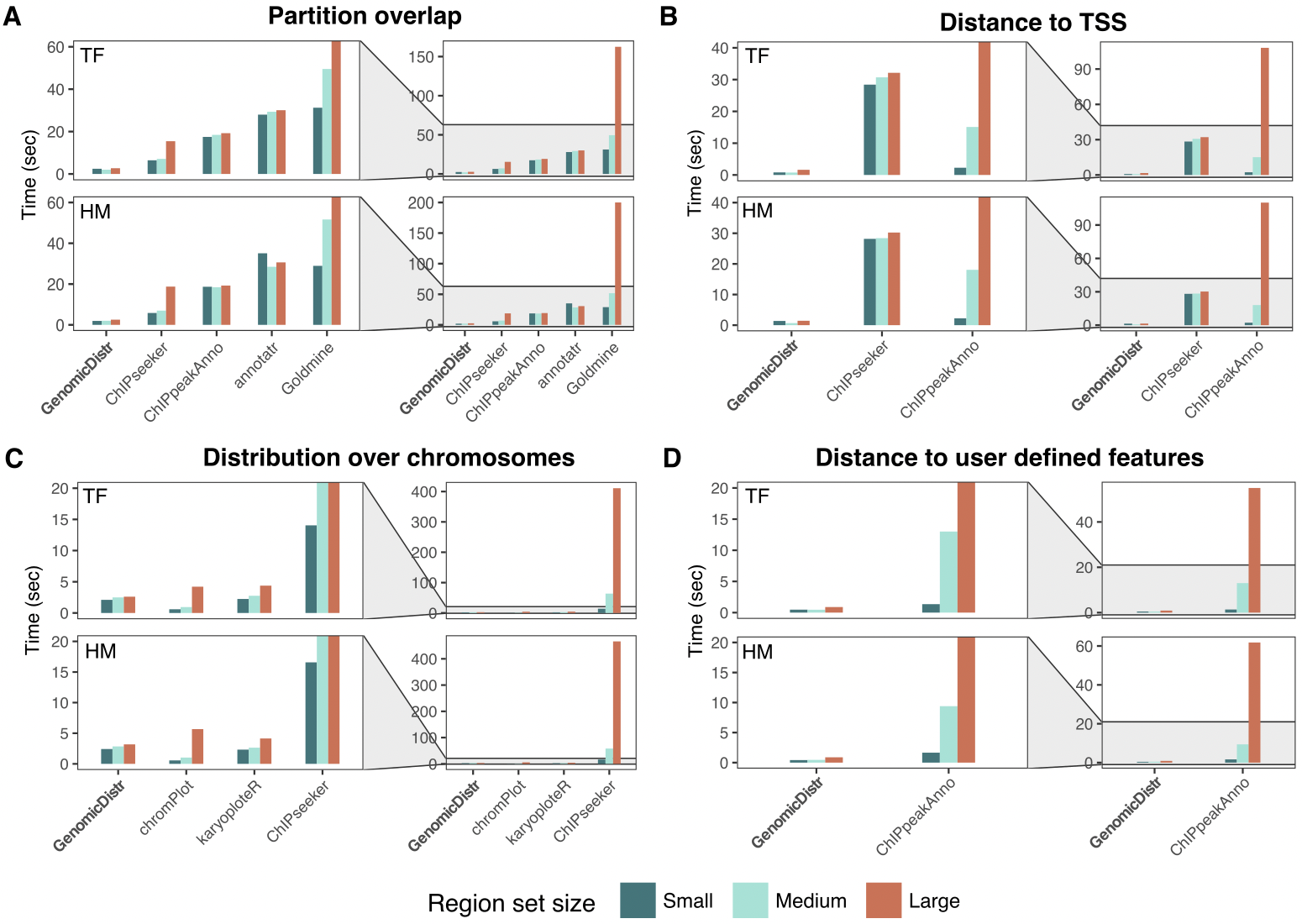


**Fig. S1. Running time benchmark for GenomicDistributions against other R packages with comparable functions.** Bar plot showing the measured running time for functions calculating the distribution of regions across different genomic partitions (A), the distance of regions to TSSs (B), the distribution of regions across chromosomes (C), distance of regions to user defined features (D). Bar plots are zoomed in to account for the presence of large running times. Performance across different packages was measured using a total of six ChIP-seq region sets (Table S3) showing variability in terms of total region number and width (TF and histone modifications) and setting data.table to use a single thread to control for default multi-threading behavior (Supplementary methods). GenomicDistr=GenomicDistributions; TF=transcription factor; HM=histone modification.


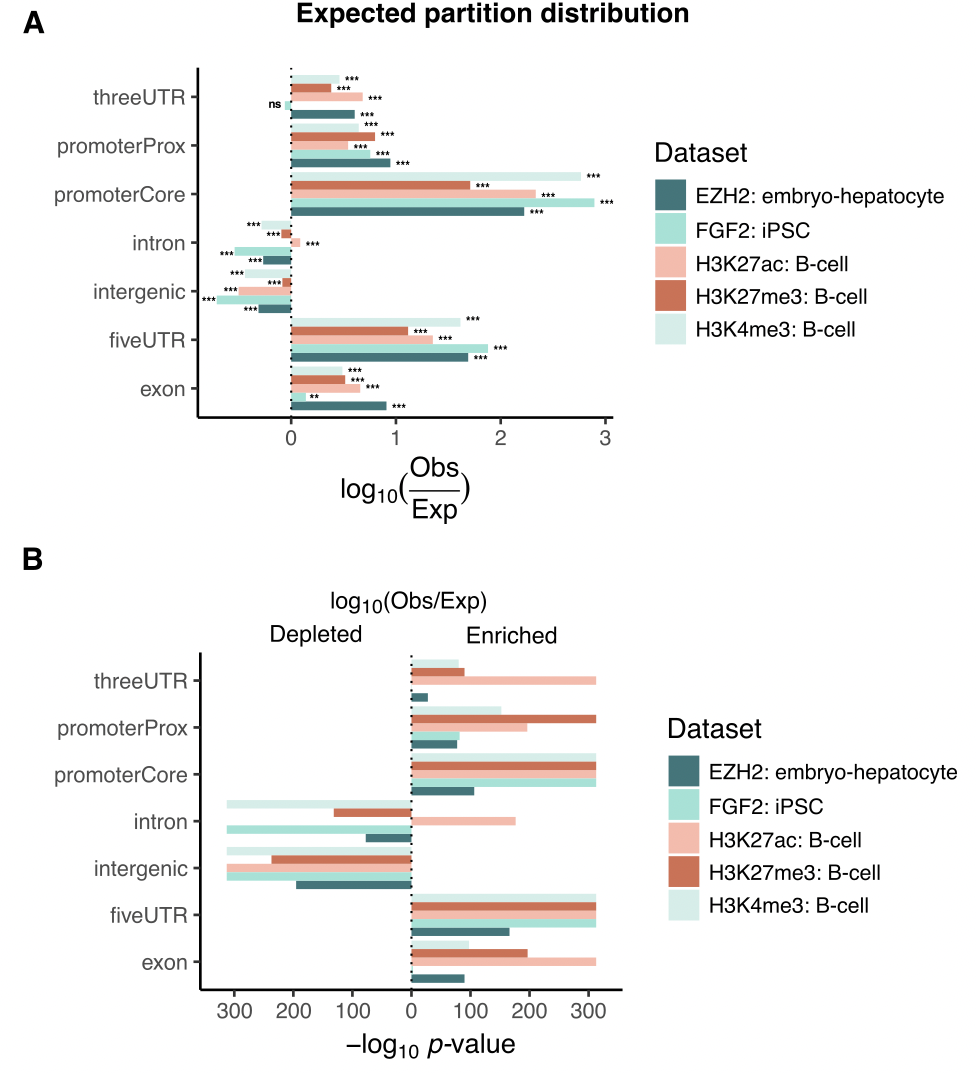


Fig. S2. Expected partition distribution plot. (A) Expected partition distribution plot corrects observed overlap frequency for the expected genome distribution. Asterisks above each bar denote the *p-*values significance levels from Chi-square tests of independence for each partition. Calculated *p-*values are detailed in Table S4. (B) Distribution of log_10_-transformed *p-*values of (A) across each partition. The direction of each bar reflects the log_10_(Obs/Exp) values for each partition denoting whether regions are enriched or depleted in comparison to expected overlaps. Asterisks denote the following: ****p* < 0.001; ** 0.001 <= *p* < 0.01; * 0.01 <= *p* < 0.05; ns *p* >= 0.05. Obs=Observed; Exp=Expected.

**
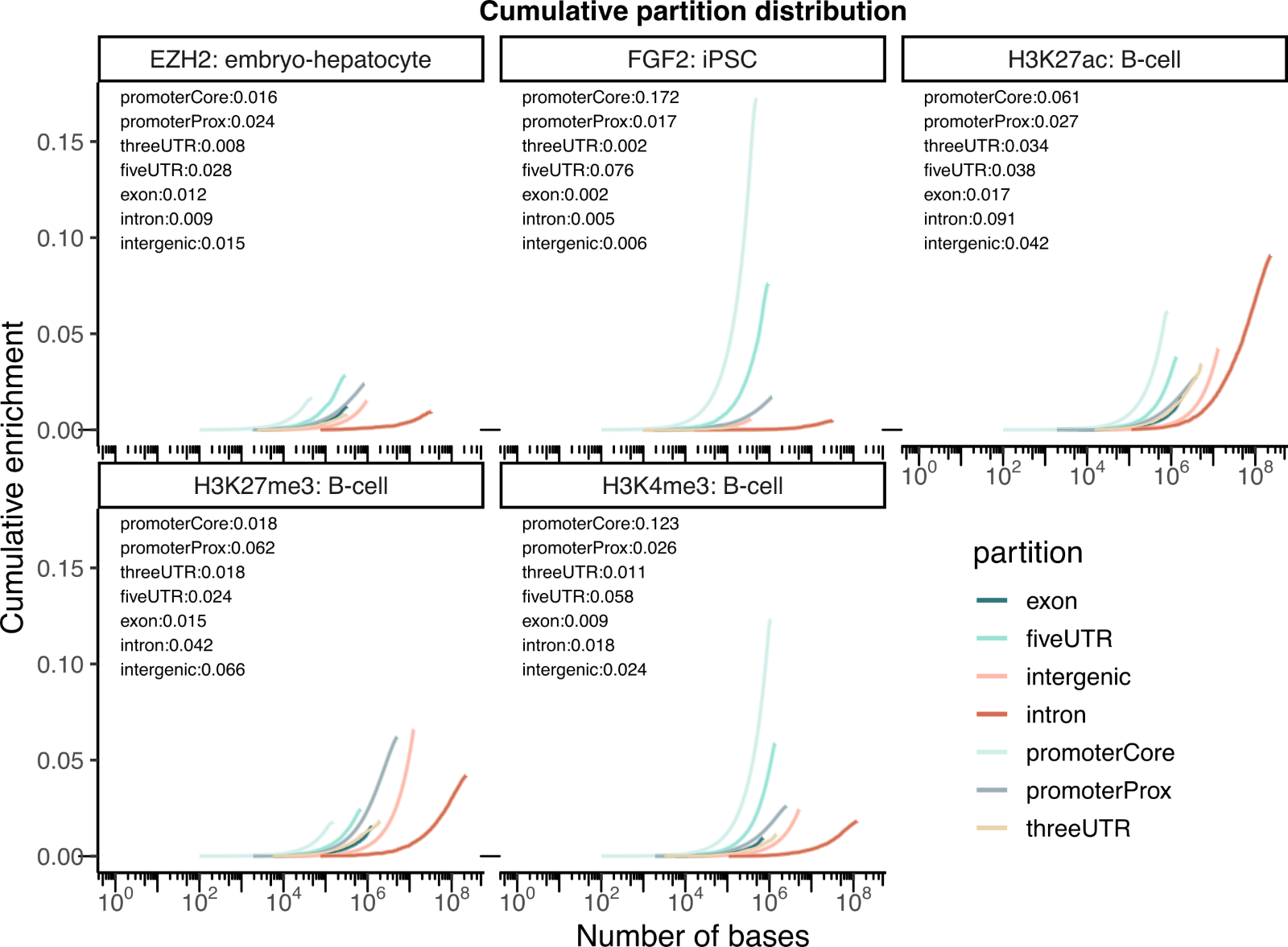
**

Fig. S3. Cumulative partition distribution plots. Plots show cumulative partition distribution curves for each region set indicated at the top of each panel. The height of each curve (y-axis) indicates the enrichment score for a given partition (see Supplementary methods: Cumulative partition plots), which is also summarized in the text in the panel. The overall width of each curve (x-axis) indicates the overall base-pair coverage of a given partition by the region set.


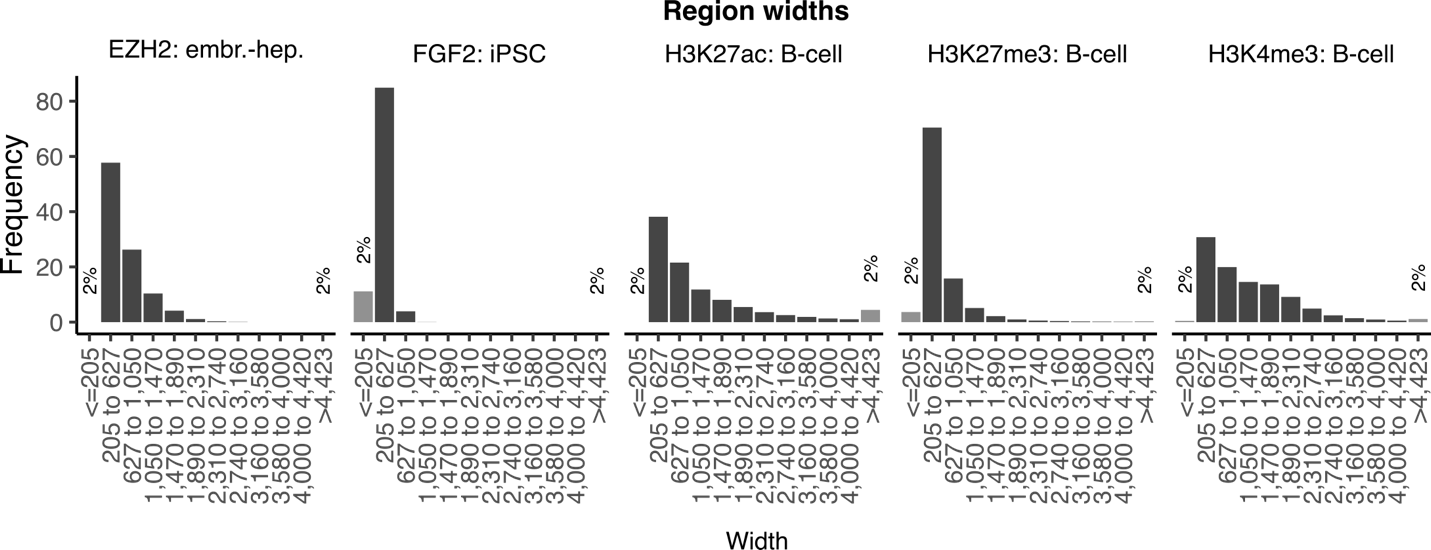


Fig. S4. Quantile trimmed region width distribution plots. Histograms showing region widths distributions with bottom and top 2 quantiles aggregated into one bin.


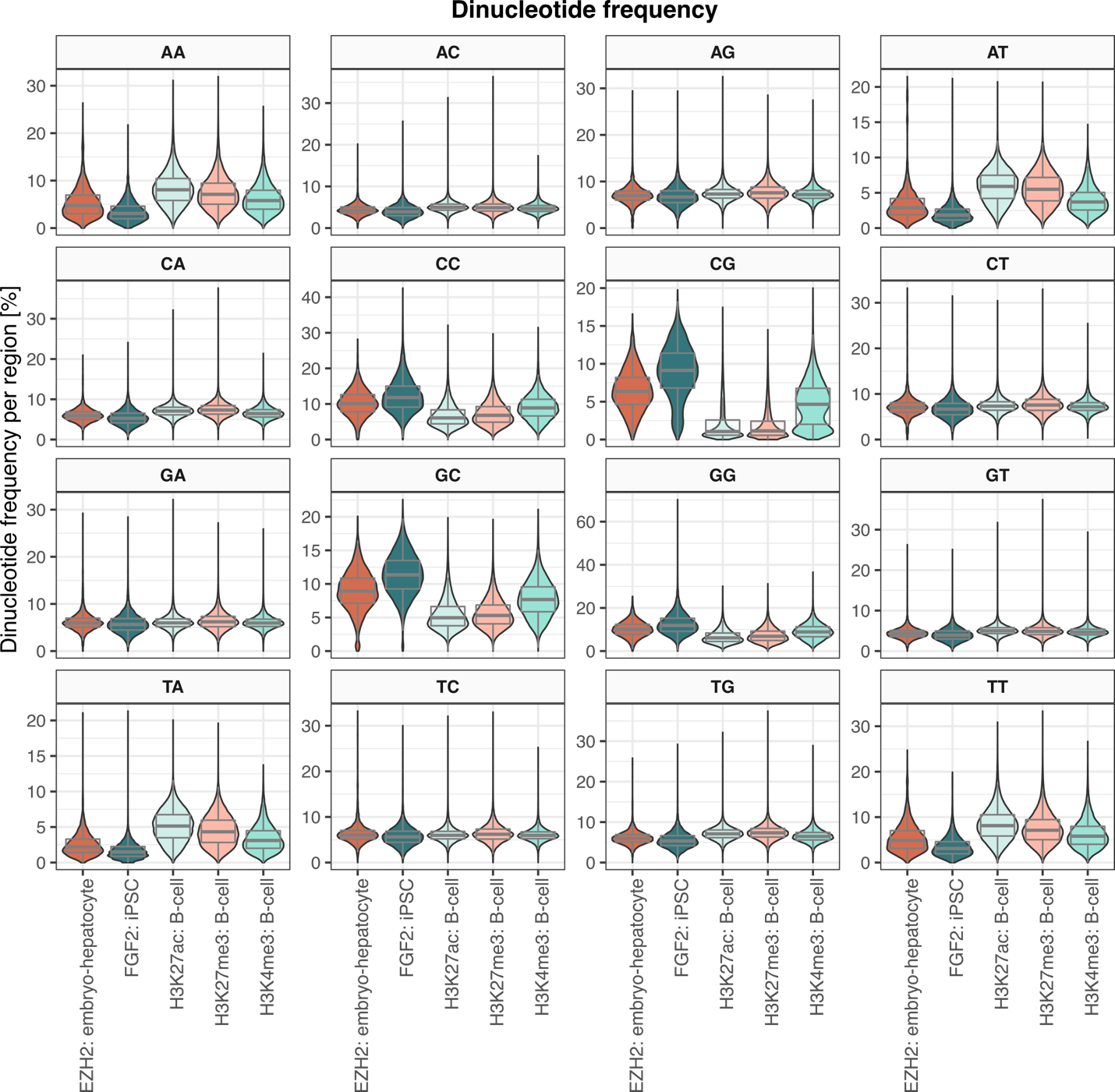


Fig. S5. Dinucleotide frequency distribution plots. Violin plots showing the distribution of dinucleotide frequency in a given region set.


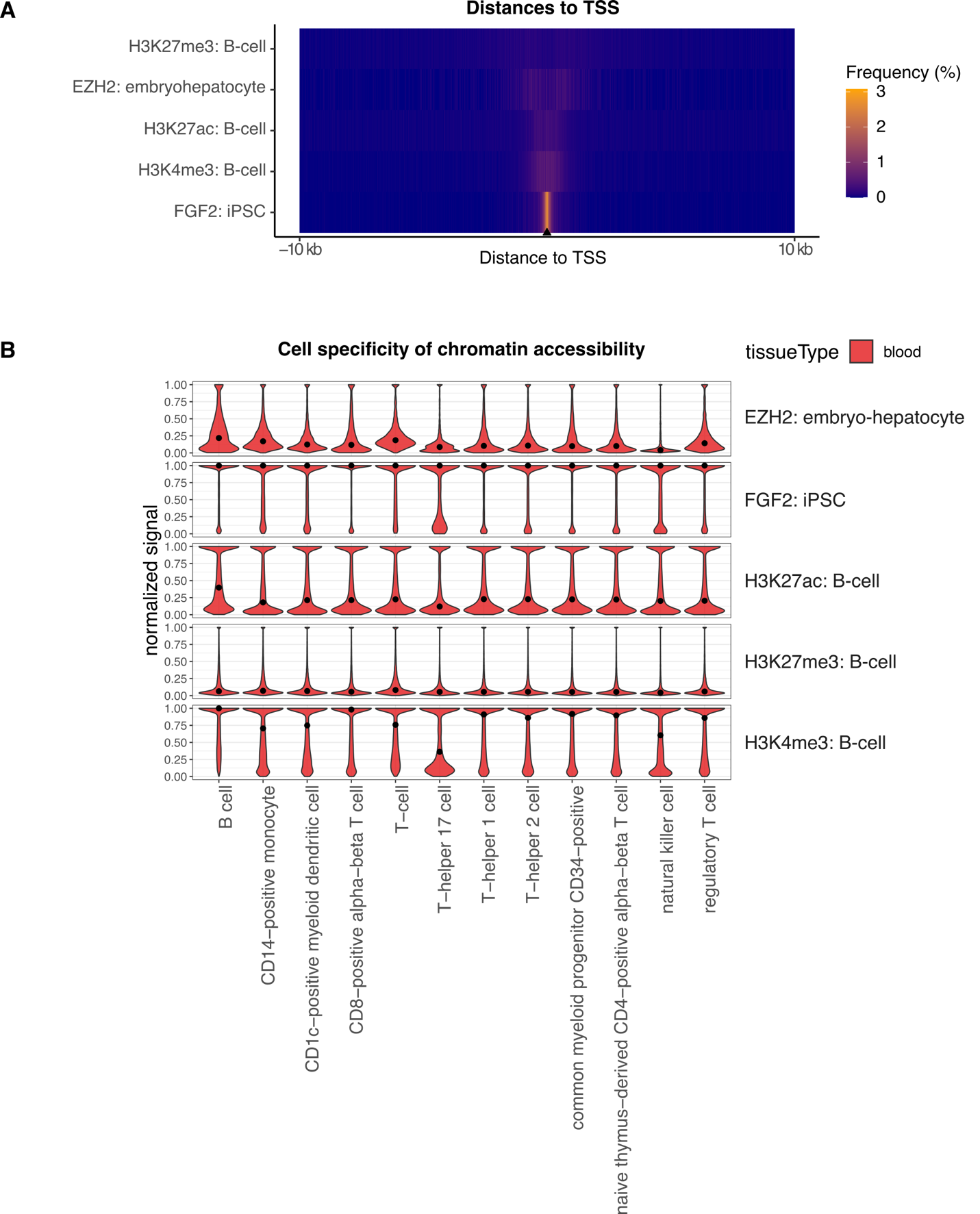


Fig. S6. Plotting enhancement options included in plotting functions. (A) Heatmap representation of distances to TSS compared to histogram representation in Fig. 2f. (B) Tissue type filter is available as part of plotting function in cell specificity of chromatin accessibility plots (Fig. 2a), as well as different plot type options such as violin plot here compared to bar plot in Fig. 2a.

# Supplementary tables

Table S1. Comparison of GenomicDistributions features to relevant tools.

|  | GenomicDistributions | rGREAT / GREAT | chromPlot | karyoploteR | ChIPpeakAnno | ChIPseeker | annotatr | Goldmine | LOLA / LOLAweb | epiCOLOC |
| --- | --- | --- | --- | --- | --- | --- | --- | --- | --- | --- |
| Distance to TSS | ✓ | ✓ | ✗ | ✗ | ✓ | ✓ | ✗ | ✗ | ✗ | ✗ |
| Distance to user defined features | ✓ | ✗ | ✗ | ✗ | ✓ | ✗ | ✗ | ✗ | ✗ | ✗ |
| Feature overlap | ✓ | ✗ | ✗ | ✗ | ✓ | ✓ | ✓ | ✓ | ✗ | ✗ |
| Chromosome distribution | ✓ | ✗ | ✓ | ✓ | ✗ | ✓ | ✗ | ✗ | ✗ | ✗ |
| Neighbor region distance | ✓ | ✗ | ✗ | ✗ | ✗ | ✗ | ✗ | ✗ | ✗ | ✗ |
| Region width | ✓ | ✗ | ✗ | ✗ | ✗ | ✗ | ✗ | ✗ | ✗ | ✗ |
| GC content | ✓ | ✗ | ✗ | ✗ | ✗ | ✗ | ✗ | ✗ | ✗ | ✗ |
| Dinucleotide content | ✓ | ✗ | ✗ | ✗ | ✗ | ✗ | ✗ | ✗ | ✗ | ✗ |
| Signal summary - e.g. cell specific signal | ✓ | ✗ | ✗ | ✗ | ✗ | ✗ | ✗ | ✗ | ✗ | ✗ |
| Multiple inputs | ✓ | ✗ | ✓ | ✓ | ✗ | ✓ | ✗ | ✓ | ✗ | ✗ |
| Plotting | ✓ | ✓ | ✓ | ✓ | ✓ | ✓ | ✓ | * | ✗/✓ | ✓ |
| Option to edit plot | ✓ | ✓/✗ | ✓ | ✓ | ✗ | ✓ | ✓ | * | ✗ | ✗ |
| Calculation separated from plotting | ✓ | ✓ | ✗ | ✗ | ✓ | ✗ | ✓ | * | ✗/✓ | ✓ |
| Gene annotation | ✗ | ✓ | ✗ | ✗ | ✓ | ✓ | ✗ | ✓ | ✗ | ✗ |
| Functional annotation - e.g. gene ontology | ✗ | ✓ | ✗ | ✗ | ✓ | ✓ | ✗ | ✗ | ✗ | ✗ |
| Region set enrichment | ✗ | ✗ | ✗ | ✗ | ✗ | ✓ | ✗ | ✗ | ✓ | ✓ |
| R / website | R | R / w | R | R | R | R | R | R | R / w | w |
| *no internal function but provided ggplot plotting instruction in vignette | | | | | | | | | | |

Table S3. *p-*values calculated from Chi-square tests for expected partition distribution calculations.

|  | ***Dataset Chi-square p-values*** | | | | |
| --- | --- | --- | --- | --- | --- |
|  | **EZH2 embryo hepatocyte** | **FGF2 iPSC** | **H3K27ac B-cell** | **H3K27me3 B-cell** | **H3K4me3 B-cell** |
| **Promoter Core** | 4.66E-107 | *p* < 1.51E-313 | *p* < 1.51E-313 | *p* < 1.51E-313 | 1.51E-313 |
| **Promoter Prox.** | 4.06E-78 | 2.87E-82 | 6.40E-197 | *p* < 1.51E-313 | 5.51E-153 |
| **Three UTR** | 1.60E-28 | 2.97E-01 | *p* < 1.51E-313 | 1.43E-90 | 9.09E-81 |
| **Five UTR** | 9.25E-167 | *p* < 1.51E-313 | *p* < 1.51E-313 | *p* < 1.51E-313 | *p* < 1.51E-313 |
| **Exon** | 1.45E-90 | 3.75E-03 | *p* < 1.51E-313 | 1.95E-197 | 3.00E-98 |
| **Intron** | 3.86E-78 | *p* < 1.51E-313 | 3.52E-177 | 2.89E-132 | *p* < 1.51E-313 |
| **Intergenic** | 5.43E-196 | *p* < 1.51E-313 | *p* < 1.51E-313 | 9.22E-238 | *p* < 1.51E-313 |

Table S4. Number of regions and mean region width of datasets used in benchmark.

| **Region set** | **Number of regions** | **Mean region width** | **ChIP-seq region set size classification** | **ChIP-seq region set width classification** |
| --- | --- | --- | --- | --- |
| TCF12 H1-hESC | 9211 | 251 | Small | TF |
| H3K27me3 H9 hepatocyte | 9884 | 1217 | Small | HM |
| ATF3 K562 | 44608 | 254 | Medium | TF |
| H3K4me3 tibial artery | 40929 | 1363 | Medium | HM |
| MEF2C GM12878 | 300000 | 303 | Large | TF |
| H3K4me1 GM23338 | 316729 | 1047 | Large | HM |

#

# Supplementary references

1. Davis CA, Hitz BC, Sloan CA, Chan ET, Davidson JM, Gabdank I, et al. The Encyclopedia of DNA elements (ENCODE): Data portal update. Nucleic Acids Res. 2018 Jan 1;46(D1):D794–801.

2. Kent WJ, Zweig AS, Barber G, Hinrichs AS, Karolchik D. BigWig and BigBed: enabling browsing of large distributed datasets. Bioinformatics. 2010 Sep 1;26(17):2204–7.

3. UCSC Genome Browser Home [Internet]. [cited 2021 Apr 5]. Available from: https://genome.ucsc.edu/index.html

4. Quinlan AR, Hall IM. BEDTools: a flexible suite of utilities for comparing genomic features. Bioinformatics. 2010 Mar 15;26(6):841–2.

5. Mersmann O. microbenchmark: Accurate Timing Functions. [R package] 2021 Nov 9. Available from: https://CRAN.R-project.org/package=microbenchmark

6. Mei S, Qin Q, Wu Q, Sun H, Zheng R, Zang C, et al. Cistrome Data Browser: A data portal for ChIP-Seq and chromatin accessibility data in human and mouse. Nucleic Acids Res. 2017 Jan 1;45(D1):D658–62.

7. Zheng R, Wan C, Mei S, Qin Q, Wu Q, Sun H, et al. Cistrome Data Browser: Expanded datasets and new tools for gene regulatory analysis. Nucleic Acids Res. 2019 Jan 8;47(D1):D729–35.
